# Effects of a Single Dose of a Creatine-Based Multi-Ingredient Pre-workout Supplement Compared to Creatine Alone on Performance Fatigability After Resistance Exercise: A Double-Blind Crossover Design Study

**DOI:** 10.3389/fnut.2022.887523

**Published:** 2022-06-21

**Authors:** Massimo Negro, Giuseppe Cerullo, Simone Perna, Matteo Beretta-Piccoli, Mariangela Rondanelli, Giorgio Liguori, Hellas Cena, Stuart M. Phillips, Corrado Cescon, Giuseppe D’Antona

**Affiliations:** ^1^Centro di Ricerca Interdipartimentale nelle Attività Motorie e Sportive (CRIAMS) – Sport Medicine Centre, University of Pavia, Voghera, Italy; ^2^Department of Movement Sciences and Wellbeing, University of Naples Parthenope, Naples, Italy; ^3^Department of Biology, College of Science, University of Bahrain, Sakhir, Bahrain; ^4^Rehabilitation Research Laboratory 2rLab, Department of Business Economics, Health and Social Care, University of Applied Sciences and Arts of Southern Switzerland, Manno, Switzerland; ^5^Department of Public Health, Experimental and Forensic Medicine, University of Pavia, Pavia, Italy; ^6^Istituto di Ricovero e Cura a Carattere Scientifico (IRCCS) Mondino Foundation, Pavia, Italy; ^7^Clinical Nutrition and Dietetics Service, Unit of Internal Medicine and Endocrinology, Istituti Clinici Scientifici (ICS) Maugeri Istituto di Ricovero e Cura a Carattere Scientifico (IRCCS), University of Pavia, Pavia, Italy; ^8^Exercise Metabolism Research Group, Department of Kinesiology, McMaster University, Hamilton, ON, Canada

**Keywords:** surface electromyography, fatigue, muscle strength and muscle power, creatine, β-alanine, glutamine, arginine, taurine

## Abstract

**Background:**

This study aims to investigate the acute effects of a single oral administration of a creatine-based multi-ingredient pre-workout supplement (MIPS) on performance fatigability and maximal force production after a resistance exercise protocol (REP).

**Methods:**

Eighteen adult males (age: 23 ± 1 years; body mass: 76.4 ± 1.5 kg; height: 1.77 ± 0.01 m) were enrolled in a randomized, double-blind, crossover design study. Subjects received a single dose of a MIPS (3 g of creatine, 2 g of arginine, 1 g of glutamine, 1 g of taurine, and 800 mg of β-alanine) or creatine citrate (CC) (3 g of creatine) or a placebo (PLA) in three successive trials 1 week apart. In a randomized order, participants consumed either MIPS, CC, or PLA and performed a REP 2 h later. Before ingestion and immediately after REP, subjects performed isometric contractions of the dominant biceps brachii: two maximal voluntary contractions (MVCs), followed by a 20% MVC for 90 s and a 60% MVC until exhaustion. Surface electromyographic indices of performance fatigability, conduction velocity (CV), and fractal dimension (FD) were obtained from the surface electromyographic signal (sEMG). Time to perform the task (TtT), basal blood lactate (BL), and BL after REP were also measured.

**Results:**

Following REP, statistically significant (*P* < 0.05) pre–post mean for ΔTtT between MIPS (−7.06 s) and PLA (+0.222 s), ΔCV slopes (20% MVC) between MIPS (0.0082%) and PLA (−0.0519%) and for ΔCV slopes (60% MVC) between MIPS (0.199%) and PLA (−0.154%) were found. A pairwise comparison analysis showed no statistically significant differences in other variables between groups and condition vs. condition.

**Conclusion:**

After REP, a creatine-enriched MIPS resulted in greater improvement of sEMG descriptors of performance fatigability and TtT compared with PLA. Conversely, no statistically significant differences in outcomes measured were observed between CC and PLA or MIPS and CC.

## Introduction

Multi-ingredient pre-workout supplements (MIPS) have attracted great interest among athletes, active people, and researchers in recent years (1). Generally, MIPS include several ingredients (e.g., caffeine, creatine, β-alanine, or other amino acids), and they promise to increase athletic performance when ingested before exercise, in a single-dose administration modality (one-shot) and without requiring a loading phase (2, 3). Notwithstanding this potential, the typical comparison design used to study MIPS in sports nutrition represents a fundamental flaw that largely limits the current knowledge on the real effectiveness, if any, of MIPS. All available trials are structured to compare the effects of MIPS vs. placebo (PLA) (1), without providing data on the direct comparison of MIPS vs. the known effective primary compound it contains (e.g., caffeine, creatine, or β-alanine) (1). Hence, this approach only considers the overall efficacy of a MIPS on the assessed outcomes, without giving any information on “what does what” within the MIPS (4). Furthermore, one-shot studies on the most of primary compounds are poorly available or completely missing, and trials on this topic should be properly addressed to acquire necessary data for single-dose comparative design studies on MIPS. For example, despite creatine is recognized as one of the most popular MIPS ingredients and included in at least 49% of the top 100 selling MIPS products (5), its efficacy in one-shot administration is unknown (6), while only a single-dose study on β-alanine (7) is currently available in the literature.

One promising field of application of MIPS regards their potential efficacy in counteracting the manifestations of exercise-induced fatigue (5) following repeated high-intensity exercise. Despite this interesting hypothesis, few studies are available on the effects of MIPS (containing creatine and β-alanine, taurine, arginine, and glutamine) on perceived fatigability, with mixed results (8–12). In contrast, data on the single-dose effects of MIPS on performance fatigability are currently unavailable. According to Enoka and Duchateau (13), *performance fatigability* is typically quantified as the decline in an objective measure of performance over a discrete period, while *perceived fatigability* refers to changes in the sensations that regulate the integrity of the performer. Many factors interact and contribute to performance fatigability (14–16). These factors may be classified as central or peripheral. The increase in the number of recruited motor units (MUs) and modulation of their discharge rate, due to a combination of intrinsic motoneuronal properties and reduction in voluntary activation of the muscle, and the increase in MU synchronization, characterize the central factors. In contrast, peripheral factors, which impair the execution of the descending central commands (17), include local changes at the skeletal muscle level that occur during prolonged activity, such as glycogen depletion, impaired regulation of calcium ions (Ca^2+^) flux in the cell matrix (18), reduction of intramuscular adenosine triphosphate (ATP) and creatine level (19), elevated concentrations of inorganic phosphate (P_i_), and excess of hydrogen ions (H^+^) (20). Even though no gold standard to assess performance fatigability is currently recognized, changes in the myoelectric activity of a selected muscle by surface electromyography (sEMG) may be used to indirectly assess performance fatigability (14). In particular, during isometric tasks, fatigue-related changes in the sEMG signal are linked to several physiological factors, such as a decay in muscle fiber conduction velocity (CV) of motor unit action potentials (MUAPs), an increase in the degree of synchronization between the firing times of simultaneous MUs (by the central nervous system), and a reduction of the recruitment threshold of MUs firing rate (21). Therefore, to assess peripheral and central components of performance fatigability, rates of change (i.e., slope) of CV (21–23), and fractal dimension (FD) of the sEMG signal, which is highly reliable on MUs synchronization (14, 24, 25), might be measured during isometric muscle tasks, respectively.

Based on the above, the aim of the this study was to evaluate the effect of a single bout of ingestion of a creatine-based MIPS on performance fatigability, measured by sEMG indices (CV and FD slopes) and maximal voluntary contraction (MVC), after a fatiguing protocol, in comparison with creatine alone and PLA. We hypothesized that MIPS ingestion could improve performance fatigability more than creatine alone or PLA, due to synergistic effects between creatine and β-alanine.

## Materials and Methods

### Participants

Eighteen trained men [age: 23 ± 1 years; body mass (BM): 76.4 ± 1.5 kg; height (H): 1.77 ± 0.01 m] completed the study. During the recruitment phase, subjects were fully informed of the aims and potential risks of the investigation and provided their written informed consent to participate in this study, before completing a questionnaire to assess their health status. Inclusion criteria included a body composition according to the reference values proposed for velocity/power athletes (26, 27) and regular participation (for at least 2 years) in resistance training or strength sports (e.g., combat sports, weightlifting, and bodybuilding). Exclusion criteria included the presence of any medical condition, fewer than three or more than five sessions per week of training, and the use of nutritional supplements containing creatine, arginine, taurine, glutamine, β-alanine, or sodium citrate in the month before the trial.

### Body Composition Assessment

The BM, H, and body composition analysis (BCA) of each participant were measured during the recruitment phase (to satisfy the inclusion criteria) and on the day before the first trial (to verify that all subjects had maintained the anthropometric characteristics at the beginning of the experimental procedures). BM and H were obtained using a mechanical scale stadiometer (Seca Ltd., Hamburg, Germany) to the nearest 0.1 kg and 0.01 m, respectively. Body mass index (BMI) was calculated as BM (kg) divided by squared stature (m). BCA was performed using a bioelectric impedance analysis (BIA EFG, Akern, Florence, Italy) following the manufacturer’s instructions (e.g., subjects lay in supine position with limbs extended away from the trunk). Electrodes (BIATRODES, Akern, Florence, Italy) were placed conventionally on the right side of the body (metacarpal lines and wrist; metatarsal lines and ankle). The physical characteristics of the subjects are illustrated in Table 1.

**TABLE 1:** Physical characteristics of the participants (*n* = 18).

Anthropometrics	Mean ± SD
Age (years)	23 ± 1
Height (m)	1.77 ± 0.01
BM (kg)	76.4 ± 1.5
BMI (kg/m^2^)	24.3 ± 0.5
FM (kg)	11.2 ± 0.7
FFM (kg)	65.2 ± 1.0

*Values are mean ± standard deviation (SD). BM, body mass; BMI, body mass index; FM, fat mass; FFM, fat-free mass.*

### Trial Organization

**FIGURE 1:**
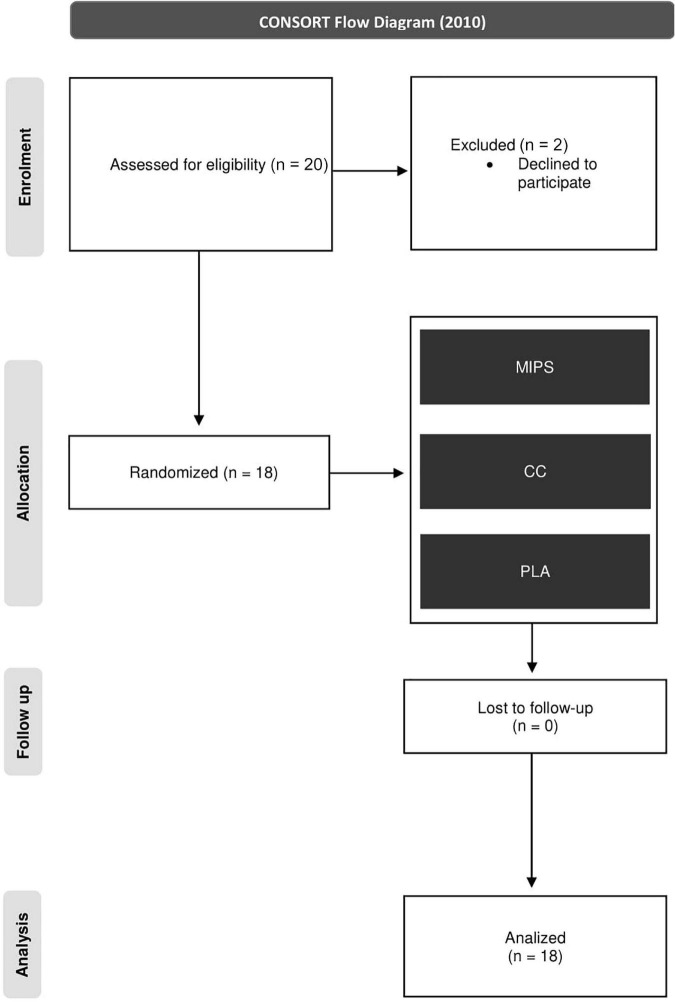
CONSORT chart.

The recruitment phase took place between September and November 2019. Potential participants were contacted first by enrollment meetings and then involved in one-to-one interviews at our medical facility. The study was conducted in accordance with the Declaration of Helsinki, and the protocol was approved by the Ethics Committee of the Department of Internal Medicine and Medical Therapy at the University of Pavia with the code 001A0012. All subjects completed all experimental procedures and measurements between November and December 2019. During the study, the dietary habits of participants were assessed and modified to keep an equal content of protein (∼1.8 g/kg/day). Furthermore, a defined daily amount of creatine-based foods (meat and fish) was recommended to achieve a similar creatine introduction in all subjects. The sample size was determined based on a previous study by Negro et al. (28), that used a similar number of subjects. Accordingly, the sample size was calculated as 18 subjects using a two-sided two-sample *t*-test at 0.05 level of significance, taking into consideration a dropout rate of 10 and 90% power. A total of 20 healthy trained males were identified and recruited for the study. Two subjects declined to participate before starting the experimental phase, and, thus, 18 subjects were analyzed. Full details of participants’ enrollment can be found in the flow diagram as shown in Figure 1. All trial phases were conducted at CRIAMS-Sport Medicine Centre, University of Pavia, located in Voghera, Italy.

### Study Design and Experimental Procedures

**FIGURE 2:**
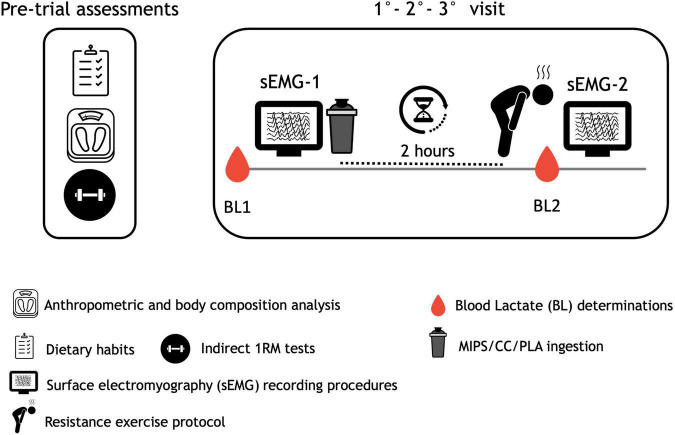
Schematic illustration of experimental procedures.

This study was a double-blind, randomized, crossover PLA and creatine-controlled trial. Subjects received a MIPS or creatine citrate (CC) or isocaloric PLA in three consecutive trials, each separated by a 1 week of washout. Specifically, they were randomly assigned, according to a computer-generated allocation schedule, to 1 of 6 treatment sequences, namely, MIPS/CC/PLA, MIPS/PLA/CC, CC/MIPS/PLA, CC/PLA/MIPS, PLA/CC/MIPS, or PLA/MIPS/CC. In each experimental trial, subjects were refrained from strenuous physical activity and were instructed to avoid drinking alcohol, coffee, or similar beverages containing caffeine or any other kind of stimulant substance in the previous 24 h. On the day of the experiment, the participants consumed a standardized breakfast (consisting of 2 rusks and 25 g of jam, with a decaffeinated tea or juice) at their home 2 h before they arrived at the laboratory (at 9:00 am). A kit containing rusks, jam, and decaffeinated tea or juice was previously provided to each subject. The three consecutive trials (i.e., 1°-2°-3° visit) consisted each of the following steps, illustrated in Figure 2: (1) basal blood lactate (BL1) determination at rest; (2) first surface electromyography (sEMG-1) recording; (3) supplement ingestion (MIPS, CC) or PLA; (4) 2 h later, a resistance exercise protocol (REP) was carried out; (5) post-REP blood lactate (BL2) evaluation; and (6) second surface electromyography (sEMG-2) recording. A similar simulation of the experimental protocol was performed 1 week before the first trial to allow the volunteers to familiarize themselves with all the procedures and avoid a “learning effect.” The reliability of the sEMG outcomes considered (CV and FD) was determined in previous studies (29, 30). Furthermore, the outcomes measured and the study design were the same as those we previously used in our lab (28).

### Resistance Exercise Protocol

To elicit fatigue in the biceps brachii, a REP was developed. It included a Wingate exercise (warm-up + 1 × 30 s) performed on an arm ergometer (Rehab Trainer 881E, Monark, Vansbro, Sweden) with a load equivalent to 5% of BM (31), followed by three sets of biceps curls with a dumbbell (Technogym, Gambettola, Italy) until exhaustion. The dumbbell load was set at 70% of 1RM, with a 90 s rest between sets. The execution time of REP was 370 ± 13.5 s in total. An indirect 1RM test was performed in all participants to set the dumbbell load (using Brzycki’s equation). The 1RM test was carried out 1 week before the first trial, to prevent any test-related muscle damage interfering with muscle performance. Completing the REP, all the participants experienced fatigue. The specific outcomes measured during REP were Wingate round per minute (WRPM), that is, the number of pedal revolutions measured during the test and expressed per minute, and biceps curl repetitions (BCRs).

### sEMG and Force Measurements

Before and after REP, the same sEMG recording protocol was used (sEMG-1 and sEMG-2). To isolate the action of the biceps brachii, the participants’ dominant arm was fixed in an isometric ergometer (MUC1, OT Bioelettronica, Turin, Italy) equipped with a load cell (CCT Transducer, linear, full scale 100 kg). Participants were seated on a height-adjustable chair with their elbows flexed at 120°. To achieve pure MUAPs propagation, according to Barbero et al. (32), a 64-electrode bidimensional array (10 mm interelectrode spacing, 8 × 8 grid) was placed on the biceps brachii, between the distal tendon and the innervation zone, on the muscle belly, previously located by an ultrasound scan (Phillips CX-30). The isolation of the muscle contraction, fluency of movement, and fiber direction made the biceps brachii the best candidate for obtaining high-quality sEMG data. The sEMG signals were amplified and sampled at a rate of 2048 Hz (EMG-USB2+, OT Bioelettronica, Turin, Italy). The setup of sEMG recording procedures was illustrated in a similar previous study realized by our lab (28).

### Performance Fatigability

Following REP, two isometric MVCs were performed after 5 min of rest, separated by a 2-min of rest. Participants were verbally encouraged to increase the force as much as possible and hold it as stable as possible for 2–3 s. Following a 2-min rest, a 90-s low-intensity contraction (20% MVC) was performed. Following a 4-min rest, participants were asked to perform a high-intensity sustained contraction (60% MVC) until exhaustion, during which they were encouraged to maintain the force level as long as possible until the force value decreased to below 5% of the target value (33). The time to perform the task (TtT) was recorded at 60% of MVC.

### Supplementation Protocol

Participants ingested either a creatine-based MIPS (3 g creatine, 2 g arginine, 1 g glutamine, 1 g taurine, and 0.8 g β-alanine) (CITROGEN–Syform, Piavon di Oderzo, Italy) or CC (4.6 g, corresponding to 3 g of creatine) (CITROFOS–Syform, Piavon di Oderzo, Italy) or a PLA (aspartame-based artificial sweetener powder). CITROGEN and CITROFOS are commercially available products containing the same amount of creatine per dose. The creatine content in each product agrees with those suggested in studies that underline as a minimum dose of 3 g is required in an MIPS to exert an ergogenic effect (2, 34). To guarantee the blindness during each step of the experimental design, MIPS, CC, and PLA powder were undistinguishable for flavor, color, and odor, and all the sachets, packaged by Akela S.r.l. (Akela S.r.l., Casier, Italy), were externally anonymous and labeled only with code numbers (CITROGEN: 19354A; CITROFOS: 19287A; PLA: 190113). MIPS, CC, or PLA were diluted in 250 ml of water and administrated in a double-blind fashion using an unmarked sports drink bottle. To avoid changes in insulin blood level, affecting energy metabolism, an artificial sweetener was used instead of a carbohydrate-based PLA.

### Signal Processing

The channels used for CV estimation were chosen based on visual inspection of individual differential signals, along with an array column, as previously described (29). Initial CV value (m/s) was estimated using a multichannel algorithm (35), on single differential signals based on matching spatially and temporally filtered signals, using non-overlapping epochs of 0.5 s. CV values outside the physiological range (3–6 m/s) were removed from the analysis (36). FD was computed on each selected signal epochs and averaged over all selected channels. The box counting method was used to estimate the FD initial value (37). Essentially, a grid of square boxes was used to cover the signal, and the number of boxes through which the sEMG waveform passed was collected. The number of boxes required to cover the signal increased exponentially when the side of the boxes was reduced in a dichotomic process. The exponential relationship was linearized when the logarithm of the number of boxes counted (log N) was plotted against the logarithm of the inverse of the box size (log 1/S). FD is the slope of the interpolation line (as determined by the least mean squared method). Performance fatigability was indirectly calculated as the slopes (% of rate of change) of the considered sEMG variables (CV and FD) (14).

### Blood Lactate Measurement

A blood sample was obtained from the earlobe, and BL concentration was determined by a specific lactate detection device (Lactate Pro 2, Arkray, Kyoto, Japan).

### Statistical Analysis

The mean and standard deviation (SD) for each variable were calculated. According to the Shapiro–Wilk normality tests, not all variables had a normal distribution, so a non-parametric pairwise test was run to compare these variables. One-way ANOVA on ranks was assessed. Pairwise analysis based on the Holm–Bonferroni *P*-value was performed to evaluate the changes in mean difference effect of treatment vs. treatment. The relationships between mean change differences among the variables were investigated using the Spearman correlation coefficient (*r*). The correlation coefficient through the linear regression analysis (*R*^2^)] was assessed to describe the strength of the associations. The *P* was calculated to assess the effect of time on the interest outcomes. Statistical analysis was performed using Statistical Package for Social Sciences (SPSS), Version 21.0 (SPSS Inc., Chicago, IL, United States), and significance was set at *P* < 0.05.

## Results

**FIGURE 3:**
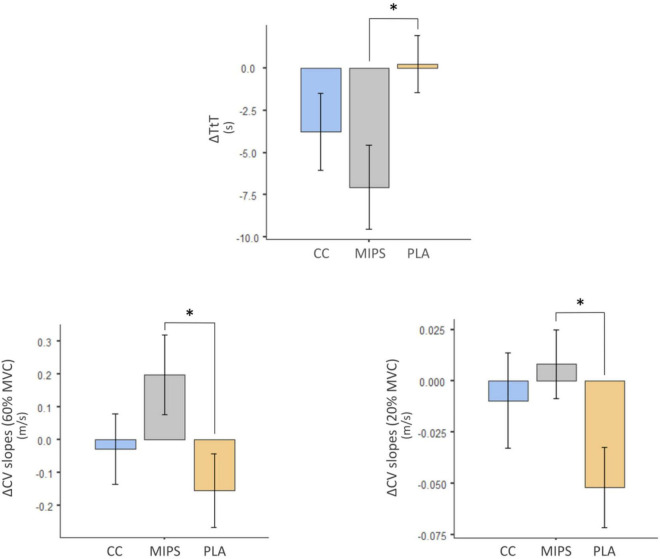
*Pre–post statistically significant mean differences for ΔTtT, ΔCV slopes (20% MVC), and for ΔCV slopes (60% MVC) between MIPS and PLA. CV, conduction velocity (m/s); CV slopes (%/s); MVC, maximal voluntary contraction (kg); TtT, time to perform the task (s).

Table 2 reports the pre–post mean differences for all outcomes investigated in MIPS, CC, and PLA. Table 3 reports treatment pairwise comparison analysis “condition vs. condition” on pre–post mean differences of Δ values of the outcomes investigated. Data show pre–post statistically significant mean differences for ΔTtT between MIPS (−7.06 s) and PLA (+0.222 s) (*P* = 0.02), ΔCV slopes (20% MVC) between MIPS (0.0082%) and PLA (−0.0519%) (*P* = 0.03) and for ΔCV slopes (60% MVC) between MIPS (0.199%) and PLA (−0.154%) (*P* = 0.03) (Figure 3). The pairwise comparison analysis recorded no other statistical differences between groups and condition vs. condition.

**TABLE 2:** Pre-post mean differences and standard error mean (SEM) among different treatments on the investigated outcomes. Values are expressed as Δ mean ± SEM.

Outcomes	Treatment	Δ Mean ± SEM
ΔTtT		
	CC	−3.76 ± 2.27
	MIPS	−7.06 ± 2.48
	PLA	0.222 ± 1.68
ΔBL		
	CC	3.79 ± 0.375
	MIPS	3.53 ± 0.416
	PLA	3.26 ± 0.379
ΔMVC		
	CC	−3.2 ± 0.475
	MIPS	−2.7 ± 0.426
	PLA	−3.08 ± 0.417
ΔFD slopes (20% MVC)		
	CC	−0.0161 ± 0.00409
	MIPS	−0.0122 ± 0.00389
	PLA	−0.00677 ± 0.00362
ΔCV slopes (20% MVC)		
	CC	−0.00942 ± 0.0234
	MIPS	0.0082 ± 0.0167
	PLA	−0.0519 ± 0.0197
ΔFD slopes (60% MVC)		
	CC	−0.0176 ± 0.0122
	MIPS	−0.025 ± 0.0136
	PLA	0.0126 ± 0.0118
ΔCV slopes (60% MVC)		
	CC	−0.0276 ± 0.108
	MIPS	0.199 ± 0.121
	PLA	−0.154 ± 0.113
WRPM		
	CC	214 ± 6.09
	MIPS	209 ± 6.00
	PLA	213 ± 5.38
BCRs		
	CC	8.2 ± 0.719
	MIPS	8.11 ± 0.664
	PLA	6.97 ± 0.42

*BCRs, biceps curls repetitions; BL, blood lactate concentration (mmol/L); CV, conduction velocity (m/s); FD, fractal dimension; CV and FD slopes (%/s); MVC, maximal voluntary contraction (kg); TtT, time to perform the task (s); WRPM, Wingate round per minute.*

**TABLE 3:** Treatments pairwise comparison analysis “condition vs. condition” on pre–post mean differences of Δ values of the outcomes investigated.

Outcomes	Treatment	Mean difference	SE	df	*t*	*P*-ANOVA	*P*-Holm
**Δ TtT**							
CC	MIPS	3.29	3.08	50	1.07	0.065	0.2072
CC	PLA	−3.99	3.08	50	−1.3		0.2708
MIPS	PLA	−7.28*	3.03	50	−2.4		0.0205
**Δ BL**							
CC	MIPS	0.26	0.556	50	0.47	0.64	1.000
CC	PLA	0.52	0.556	50	0.95		1.000
MIPS	PLA	0.26	0.548	50	0.49		1.000
**Δ MVC**							
CC	MIPS	−0.49	0.624	50	−0.8	0.707	1.000
CC	PLA	−0.11	0.624	50	−0.19		1.000
MIPS	PLA	0.37	0.615	50	0.61		1.000
**Δ FD slopes (20% MVC)**							
CC	MIPS	−0.003	0.0055	50	−0.71	0.245	0.646
CC	PLA	−0.009	0.0055	50	−1.69		0.291
MIPS	PLA	−0.005	0.0054	50	−1		0.646
**Δ CV slopes (20% MVC)**							
CC	MIPS	−0.017	0.0284	50	−0.62	0.099	0.7139
CC	PLA	0.042	0.0284	50	1.49		0.0860
MIPS	PLA	0.060*	0.028	50	2.15		0.0389
**Δ FD slopes (60% MVC)**							
CC	MIPS	0.0073	0.0179	50	0.41	0.089	0.683
CC	PLA	−0.0302	0.0179	50	−1.69		0.194
MIPS	PLA	−0.0375	0.0176	50	−2.13		0.114
**Δ CV slopes (60% MVC)**							
CC	MIPS	−0.227	0.162	50	−1.39	0.092	0.1884
CC	PLA	0.127	0.162	50	0.78		0.3780
MIPS	PLA	0.353*	0.16	50	2.21		0.0307
**WRPM**							
CC	MIPS	5.183	8.28	50	0.63	0.80	1.000
CC	PLA	0.989	8.28	50	0.12		1.000
MIPS	PLA	−4.194	8.16	50	−0.51		1.000
**BCRs**							
CC	MIPS	0.0944	0.869	50	0.11	0.29	0.914
CC	PLA	1.2333	0.869	50	1.42		0.485
MIPS	PLA	1.1389	0.856	50	1.33		0.485

*Statistically significant value. BCRs, biceps curls repetitions; BL, blood lactate concentration (mmol/L); CV, conduction velocity (m/s); FD, fractal dimension; CV and FD slopes (%/s); MVC, maximal voluntary contraction (kg); TtT, time to perform the task (s); WRPM, Wingate round per minute.

## Discussion

The first aim of our study was to evaluate the effects of the MIPS on performance fatigability, assessed by measuring sEMG descriptors as CV and FD slopes, and the TtT (at 60% MVC to exhaustion) compared with CC and PLA. Previous studies based on similar procedures and treatments have never been published before, and thus it is not possible to directly compare our results with available data. However, we suggest that creatine-based MIPS positively affected performance fatigability (CV slopes) and TtT after REP, while no effects were observed on central factors (FD slopes). Even though MIPS was unable to significantly affect maximal force production (MVC) or performance outcomes (WRPM or BCRs), the positive effect on CV slopes and TtT registered in MIPS, but not in CC and PLA, may relate to the acute positive action of the supplement on peripheral components of performance fatigability, independently from the protons buffering function of creatine (38) and its ergogenic contribution (34). The observed results after MIPS may be linked to two possible additional mechanisms, which involve other components of the mixture, able to synergistically produce benefits to different levels of muscle work intensities, as we observed through the measure of CV slopes at 20 and 60% of MVC: (1) an acute role of β-alanine on the buffer capacity of the muscle during the fatiguing task as previously hypothesized by Invernizzi et al. (7), although we were not able to provide biochemical evidence of this assertion by measuring differences of blood pH, and (2) an overall “antifatigue” effect due to the presence of molecules able to regulate recognized mechanisms involved in the onset of high-intensity resistance exercise-induced fatigue (39–41), such as glutamine, arginine, and taurine. Glutamine is an ammonia buffering and its supplementation was associated with the prevention of ammonia accumulation (42); arginine is the precursor of nitric oxide (NO) and thus plays an important role in increasing of endothelium-dependent vasodilation and blood flow to skeletal muscle, which may impact the TtT during high-intensity isometric contractions (43); taurine has been reported to reduce resistance exercise-related fatigue by decreasing oxidative stress due to muscle damage (44).

The second aim of our study was to investigate the acute effect of ingestion of the MIPS on MVC. In our procedures, MVC was measured to detect the MVC during an isometric task and to evaluate the impact of fatigue on this type of muscle work. We discovered that neither MIPS nor CC affected MVC after REP. This result is not surprising considering that all the available studies on creatine and β-alanine supplementation suggest that 5–7 days and 4 weeks of administration are required, respectively, to increase muscle stores of their active metabolites (i.e., phosphocreatine and carnosine) and effectively increase the acute exercise capacity (34, 45). At present, only one study by Invernizzi et al. (7) provided evidence that a single bolus of β-alanine (2 g of β-alanine combined with 2 g of L-carnosine) in physically active young males was able to promote a positive effect on MVC compared with PLA, during isometric and dynamic tests performed 4 h after the supplement ingestion. In this study, the change in force production was attributed to the transient amelioration of the buffer capacity of the muscle, able to limit the drop of the MVC. However, compared with our experimental conditions, in this study (7), the β-alanine dosage was higher (2 g of β-alanine), and the time interval before the tests was longer (4 h). As regards the effects of a single acute dose of creatine supplementation, no studies are currently available, and, generally, more than 2 days of creatine loading are considered to be required to elicit significant improvement of muscle strength and power benefits (6).

### Limitations

Considering that the time of administration of supplements used in this investigation was based on the available pharmacokinetic data (46–48), a main limitation of this study is the lack of blood concentrations of metabolites related to the supplement after their ingestion. Further, an *in vitro* analysis of muscle amount and timing of compound enrichment through biopsy samples would be required to establish the optimal dose and timing of ingestion of a MIPS. Unfortunately, at present, only data from pharmacokinetic studies are available. For example, 10 mg/kg of β-alanine (equivalent to 800 mg for an individual of 80 kg) induced an increase of plasma β-alanine values that peaked 40 min after ingestion (46). Similarly, blood creatine achieves peak concentrations ∼1 h after ingestion (47, 48). Based on these considerations, findings from published studies examining the acute effect of MIPS on exercise outcomes from a few minutes to less than 1 h after ingestion are questionable (8–10, 49–60). Finally, considering that BL accumulation is an indirect indicator of an increased proton release by the muscle and the potential decreased cellular, and blood pH (61), future studies regarding the buffering effects of MIPS should include direct pH measurements.

## Conclusion

To the best of our knowledge, this study established, for the first time, the effect of a single dose of a creatine-based MIPS on performance fatigability compared with creatine alone and PLA. The results indicate the effectiveness of the chosen MIPS compared with PLA to ameliorate sEMG descriptors of performance fatigability and its inefficacy in improving force generation capacity and performance outcomes when ingested before a REP. Conversely, no statistically significant differences on outcomes measured were observed between CC and PLA or MIPS and CC. Based on these findings and the available literature, we can suggest that the administration timing of a MIPS, similarly to those used in our study, should be different according to the objective to be achieved: (1) a single dose, ingested 2–4 h before resistance training, to reduce fatigue. In this case, a MIPS may be more effective than creatine alone; (2) a single or multiple doses ingested daily, depending on the intensity/duration of muscle work to be carried out, for at least 2–5 days before resistance training, to have possible effects on strength and muscle power. In this case, a multiday administration may be necessary to have a greater effect from the administration of creatine and β-alanine.

## Data Availability Statement

The raw data supporting the conclusions of this article will be made available by the authors, without undue reservation.

## Ethics Statement

The studies involving human participants were reviewed and approved by the Ethics Committee of the Department of Internal Medicine and Medical Therapy at the University of Pavia with the code 001A0012. The patients/participants provided their written informed consent to participate in this study.

## Author Contributions

MN and GD’A conceived the original idea. MN and GC performed the experiments and wrote the manuscript. CC analyzed the data. SP performed the statistical analysis. MB-P, MR, GL, HC, SMP, and GD’A revised the manuscript before submission. All authors agreed to the content of the final version of the manuscript.

## Conflict of Interest

SMP reports grants or contracts from the U.S. National Dairy Council, Dairy Farmers of Canada, Roquette Freres, Nestle Health Sciences, National Science and Engineering Research Council, and Canadian Institutes for Health Research during the conduct of the study; personal fees from U.S. National Dairy Council, non-financial support from Enhanced Recovery, outside the submitted work. In addition, SMP has a patent Canadian 3052324 issued to Exerkine, and a patent US 20200230197 pending to Exerkine but reports no financial gains from any patent or related work. None of the organizations or companies mentioned by SMP has commercial or financial relationships with Syform S.r.l. that could be construed as a potential conflict of interest in this study. The remaining authors declare that the research was conducted in the absence of any commercial or financial relationships that could be construed as a potential conflict of interest.

## Publisher’s Note

All claims expressed in this article are solely those of the authors and do not necessarily represent those of their affiliated organizations, or those of the publisher, the editors and the reviewers. Any product that may be evaluated in this article, or claim that may be made by its manufacturer, is not guaranteed or endorsed by the publisher.
